# Update on Nutrition and Food Allergy

**DOI:** 10.3390/nu14102137

**Published:** 2022-05-20

**Authors:** Nicolette W. de Jong, Harry J. Wichers

**Affiliations:** 1 Department of Internal Medicine, Section of Allergology & Clinical Immunology, Erasmus MC, University Medical Centre Rotterdam, 3015 GD Rotterdam, The Netherlands; 2 Department of Paediatric Allergology, Sophia Children Hospital, Erasmus MC, University Medical Centre Rotterdam, 3015 GD Rotterdam, The Netherlands; 3 Food & Biobased Research, Wageningen University & Research Centre, 6708 WG Wageningen, The Netherlands; harry.wichers@wur.nl

Food-induced anaphylaxis is an immediate adverse reaction, primarily triggered by the cross-linking of allergen-specific immunoglobulin (Ig) E bound to the high-affinity IgE receptor (FcεRI) on mast cells (MCs) after re-exposure to the same food allergen. Patients with an IgE-mediated food allergy often suffer from a variety of symptoms, e.g., gastrointestinal, skin, lungs, and, in the worst case, anaphylaxis. The site where food antigens are firstly taken up, either the skin or the gut, may cause sensitization against this food antigen [1]. Sensitization in the gut can take place due to increased intestinal permeability, and in the skin, a disrupted skin barrier is often responsible for higher exposure to allergens, which consequently leads to increased sensitization. This is often the case in patients with atopic dermatitis. In these patients, often 20% of the body area is affected, mostly caused by mutations in filaggrin (FLG) null, which encodes for the epidermal protein FLG. The IL-33 levels in these patients are high, mostly caused by scratching. This also increases the degranulation of mast cells and intestinal permeability. Van Splunter et al. described increased interleukin (IL)-33 levels in serum, which activate dendritic cells (DCs) and interleukin 2 (ILC2) cells [2]. Furthermore, cutaneous sensitization induces thymic stromal lymphopoietin (TSLP) activation of basophils, and the production of IL-4, IL-5 and IL-13, leading to a reduced gut barrier for food allergens and an IgE-mediated degranulation of MCs. This illustrates the existence of a skin-to-gut crosstalk, in which damaged skin can promote food-induced anaphylaxis by driving intestinal MC expansion.

Sensitization to food allergens can be measured with the Skin Prick test (SPT) and/or specific serum IgE (sIgE). Unfortunately, standardized commercial food allergen extracts for SPT are less readily available. Furthermore, due to globalization, the number of foods that causes an allergic reaction is increasing. In addition to the SPT, the Prick-to-Prick test (PTP) is also very commonly used to measure sensitization. The PTP test shows high sensitivity and specificity, but it is not very practical, as fresh fruits have to be available at the department. One alternative for commercial extracts and fresh fruits for PTP might be to prepare homemade (HM) extracts through standardized protocols. Recently, S. Terlouw et al. performed a clinical trial in 54 food-allergic patients comparing SPT results with commercial and home-made extracts [3]. Extracts form hazelnut, walnut, apple, peach and almond were compared. The intraclass correlation coefficient between the SPT results of both extract methods was strong for hazelnut, moderate for apple and peanut and weak for the other allergens. Many SPT’s with almond were positive without causing symptoms in the patients. In contrast, results with home-made peach extract showed high agreement with the peach-specific allergic symptoms. The homemade extract consists of a few drops of juice that are rapidly produced from the whole peach and stored in small aliquots at −20 °C. This method mimics the PTP method. In that way, many small aliquots from different fruits and vegetables, and even from fresh herbs, can be available every day at the clinic.

The evaluation of patients with a possible food allergy starts with an extensive food-specific medical history. The standardized diet history tool published by S. Skypala et al. provides a practical approach to support food allergy diagnosis, ensuring that all relevant information is captured and interpreted in a robust manner [4]. Although the combination of the allergy-focused diet history with positive sensitization to the specific food allergen in SPT and/or sIgE measurements often leads to a clear diagnosis, in many cases, discrepancies occur. The only method (gold standard) to finally confirm a food allergy is to perform a double-blind, placebo-controlled food challenge (DBPCFC). This is very time-consuming, expensive and at risk for anaphylaxis, but in some cases, it is indispensable and therefore commonly used. Masking the food for a DBPCFC is not always possible, and therefore, in some cases, the patient is blinded and uses a nose clip. This method is called single blinded. A good example of this kind of test is the pear study performed by de Jong et al. [5]. In the Netherlands, research has been conducted to measure differences in allergenic properties between various cultivars, e.g., pear cultivars. Also in the Netherlands, in early 2007, the “Santana apple” showed reduced allergenic properties because of its lower Mal d 1 levels. The Santana apple caused significantly fewer allergic symptoms in apple-allergic individuals than the Golden Delicious and Topaz apples [6]. Unfortunately, this lower allergenicity could not be measured for a new “Cepuna” pear cultivar. The results of challenges with the new ‘Cepuna’ pear were comparable with the more common “Conference” pear. The only (non-significant) difference in favour of the Cepuna pear was that it caused less objective symptoms and less severe symptoms after consumption. The highest dose used in the challenge was 200 g, which comes close to a whole pear. Doses and volumes of foods often lead to discussions among allergists and dieticians. In challenges, the patient reacts to a certain dose, but the question remains how to translate that to normal consumption. When the patient reacts to 100 mg of protein, is that comparable to a spoon, a bite, a sip or a cup? For the management of food allergies, we should be aware that there are no standard definitions. Recently, M. Kok et al. estimated sizes of bit and sip for milk, egg, peanut and hazelnut in selected age groups: 2–3, 4–6 and 19–30 years [7]. The results could be compared with ED10 and ED50 (10% and 50% of the allergic subjects react with objective symptoms) [8]. Only one food contained less estimated allergenic protein per portion when comparing the amount of milk in foods to the ED10 for milk. This was the case for four foods: for egg, peanut and hazelnut none of the foods contained less than the ED10. This means that all the other foods will provoke allergic reactions in allergic patients who belong to the 10% most clinically sensitive individuals. The protein content in a single bit or sip contained a sufficient amount of allergenic protein in all cases to elicit an allergic reaction.

Doses and servings become more important since the early introduction of foods is advised for the prevention of food allergy. S. Filep et al. published doses of specific allergens in “early introduction foods” (EIF) for the prevention of food allergy for 17 major food allergens [9]. Cumulative allergen doses for each EIF were estimated using serving sizes and consumption recommendations provided by the manufacturer. For early introduction of foods, as well as of introduction of foods after a negative food challenge, the doses are of high importance [10]. The starting dose for introduction after a negative food challenge should not exceed the highest dose that was given during the food challenge. In individual cases, an introduction schedule can be provided to the patient for home introduction, and in other cases, the doses should be given at the outpatient clinic. Furthermore, regular telephone calls are important to follow-up the patient. Two studies comparing introduction with and without a structured protocol showed significant differences: vd Valk et al. [11] and JAM Emons et al. [12]. The latter study showed only 8% of failed introductions versus 52% in the earlier study by Valk et al. So, protocols and follow-ups are mandatory to successfully introduce the food into the daily diet of the patient.

Since 2011, when J.S. Kim et al. [13] published a paper proving that dietary “baked milk” accelerates the resolution of cow’s milk allergy (CMA) in children, many trials have studied the effects of processing of foods, e.g., baking and drying. Apparently, (dry) heating and glycation of cow’s milk protein (Maillard reaction) have been shown to alter its digestibility and immunogenicity, and consequently, CMA children are able to consume this form of cow’s milk (CM). Moreover, “baked milk” products (using dry heating) have been shown to accelerate the resolution of cow’s milk allergy. The study of Zenker et al. Investigated specific peptide profiles of CM proteins heated at low and high temperatures after simulated infant in vitro digestion and compared this to non-treated CM [14]. This study showed that during simulated infant in vitro digestion of milk that was dry heated in the presence of lactose, different peptide profiles are generated. High-temperature dry heating had the largest effects on peptide generation, resulting in much lower numbers of peptides with lower sequence coverage. Moreover, a much lower number of sIgE-binding epitopes and a larger proportion of glycated sIgE-binding epitopes and T-cell epitopes in heated samples indicated that the immunogenicity and allergenicity of these samples could be affected.

Many studies have investigated the tolerance-inducing effect of baked milk, but the form of the product (e.g., cake, bread, cheese or pizza) and the precise heating process were found to be highly variable. For the introduction of, e.g., milk and egg, so-called milk and egg ladders can be used, but even the latest literature from Venter et al. in 2022 [15] does not give detailed information on the exact baking temperature or baking time of the products. Even protein content of the several doses is unknown. FrieslandCampina (Amersfoort, the Netherlands) developed a standardized dry-heated CM protein powder, with an exact baking temperature and time, and the method is accurately described in the article [16]. To test the new baked milk (HP) powder, challenge-proven CMA children were included (3 months–3 years), and the HP powder was introduced in incremental doses by dissolving it in the child’s daily milk formula. Seventy-two percent (18/25) of the children tolerated the HP product, and seven children experienced adverse events. These results are comparable with the baked milk studies. The group that does not tolerate the baked milk product most likely has a more severe or even a persistent CM allergy. Currently, a randomized placebo-controlled study is being carried out in 10 different children hospitals using this HP powder to measure the tolerance-inducing capacities of the product. The results are expected by the end of 2022. The Maillard reaction (MR) can affect the sensitization properties of allergens in patients. The process is widely studied in CM allergy, but studies with other food allergens can hardly be found. Wheat flour is an important component of many baked goods, and during the baking process, wheat protein may also undergo the MR because sugars are usually present. However, reports on the allergenicity change in wheat proteins after glycation are rare. This was also concluded by Gou et al. in a recent review [17]. An important allergen of wheat is gluten, especially in the form of glutenin. Methylglyoxal (MGO) has the highest reactivity as intermediate in the MR. The project of Wang et al. aimed to determine the effect of MGO on the allergenicity of glutenin based on the BALB/c mouse model pre-sensitized to native glutenin, heated glutenin and MGO-glutenin, in order [18]. The digestibility and changes in the structure of glutenin and gut microflora in mice were analysed to elucidate the detailed mechanism by which the potential for allergic reaction is reduced as a result of MGO decoration. The current research results show that glutenin could alleviate the resulting allergic reaction in mice after MGO decoration. This study provides a theoretical basis for alleviating glutenin allergic reactions through processing which should be confirmed in clinical trials in humans.

To further investigate whether food components or processed food components have effects on the adaptive immune system, intervention studies are widely suggested. The debate is still far from consensual, in particular on skewing the immune system towards a more homeostatic situation by means of inducing production of higher numbers of Tregs, which can decrease the number of T-helper 2 (TH2) cells and consequently decrease, e.g., IL-4 and IL-5 production. Lately, the supplementation of brown seaweed is presented in literature as having modulating properties on adaptive immune responses. The article by Kamunde et al. showed a highly significant increased total plasma antioxidant capacity in fish [19]. These reactive oxygen species are known to be important drivers of inflammation. Recently, E.M. Olsthorn published an extensive review on brown seafood supplementation and its effects on allergy and inflammation and their consequences [20]. They consider the seaweed effects by enhanced production of IL-1 and TNF, as well as secondary cytokines, such as IL-10. IL-10 specifically has a clear immuno-suppressive effect. Allergen immunotherapy induces IL-10-producing type 2 innate lymphoid cells, which are strongly associated with a clinical response by modulating grass pollen allergy [21]. In this light, better-designed human studies applying individual seaweed constituents, as well as whole seaweed (extracts), will provide more insight into the applicability of brown seaweed as an immune-modulatory nutritional intervention strategy.
